# Impact of group training on compassion, empathy, and stigmatizing thoughts: a diversity, equity, and inclusion pilot RCT

**DOI:** 10.3389/fpsyg.2025.1547645

**Published:** 2025-06-20

**Authors:** Sima Nurali Wolgast, Linnea Lunde-Flennmark, Anna Nestorson, Martin Wolgast, Eva Hoff

**Affiliations:** Department of Psychology, Lund University, Lund, Sweden

**Keywords:** diversity, equity, and inclusion training, anti-discrimination, compassion training, empathy, stigmatizing thoughts, prejudice

## Abstract

**Introduction:**

The purpose of this pilot study was to examine the feasibility of a compassion-based Diversity, Equity, and Inclusion (DEI) intervention. Specifically, the study aimed to investigate whether the intervention led to increases in compassion and empathy, and decreases in stigmatizing thoughts and prejudice.

**Methods:**

Participants (*N* = 33) were randomized into two groups. The experimental group received compassion training, while the comparison group received lectures on prejudice and discrimination. Measures of compassion, empathy, stigmatizing thoughts, and modern prejudice were administered pre- and post-intervention.

**Results:**

The results showed that participants in the experimental group reported increased levels of compassion and empathy, along with a tendency toward decreased stigmatizing thoughts. However, no reduction in modern prejudice was observed.

**Discussion:**

These preliminary findings suggest that compassion training may foster greater compassion and empathy, which in turn could contribute to reducing stigmatizing thoughts. Although no effect was found on modern prejudice, the results support the potential of compassion-based approaches in DEI interventions. Further research with larger samples is needed to replicate these findings and explore their applicability in organizational settings.

## Introduction

Prejudice and discrimination cause serious problems in societies worldwide, such as biased recruitment in the work market (Wolgast and Wolgast, 2021) and health injustice (Paradies et al., 2013). Despite the existence of legislation in numerous countries mandating proactive organizational efforts against discrimination, various forms of discrimination persist. Discrimination is often the result of prejudiced or stereotypical thinking; therefore, anti-discrimination work is directed toward reducing prejudice or processes influencing prejudice. The key question is why prejudice and discrimination are difficult to combat.

Contemporary prejudice, often termed “modern prejudice” (Akrami and Ekehammar, 2005), differs from traditional forms because of its less overt expression. Furthermore, individuals’ lack of awareness of how their negative thoughts about others influence their behavior presents an additional challenge (Levin et al., 2014). These subtle manifestations of attitudes and thought processes are more challenging to identify and address, potentially contributing to the continued prevalence of discrimination. What interventions can lessen stigmatizing thoughts and modern prejudice?

There is research indicating that empathy and compassion can be important mechanisms to strengthen the effects of anti-discrimination and diversity, equity, and inclusion (DEI) training, having the potential to influence the less conscious stigmatizing thoughts and emotional aspects of prejudice. Meta-studies have found that empathy and perspective-taking training have effects on negative outgroup attitudes (Beelmann and Heinemann, 2014; Paluck et al., 2021), and one study indicated that self-compassion and compassion can mitigate negative intergroup attitudes (Vu and Rivera, 2023). Empathy refers to both an emotional aspect, the ability to feel sympathy for another, and a cognitive aspect, the ability to adapt and understand another person’s point of view (Davis, 1983). Compassion is defined as “sensitivity to suffering in self and others, with a deep commitment to try to relieve it” (Gilbert, 2009, p. 3).

There are some very brief interventions with adults demonstrating how compassion mindfulness exercises decreased bias and discrimination (Parks et al., 2014; Lueke and Gibson, 2016), as well as one with a longer duration that reduced implicit prejudice (Kang et al., 2014). However, the mechanism of compassion was unmeasured in these studies, obscuring whether compassion strengthening or other effects drove these outcomes. Other studies with workplace samples have combined compassion content with other approaches and demonstrated a reduction in prejudice and/or stigmatizing thoughts (Wolgast et al., 2024; Ilanius Göransson et al., 2025).

These studies indicate that the integration of clinical, mindfulness, and sociopsychological approaches can be feasible in the development of new DEI methods. In our study, the integrated compassion aspects refer to the application of compassion-based training as conceptualized within clinical frameworks, particularly Compassion Focused Therapy (CFT), but also within the non-clinical framework of mindful compassion (Gilbert and Choden, 2014). Though compassion is not inherently a clinical concept, CFT offers a structured approach to cultivating compassion and reducing shame and self-criticism. By incorporating clinical and mindfulness-grounded practices into a DEI intervention for adults, we aim to bridge therapeutic mechanisms with broader social psychological goals, such as prejudice reduction.

The current study examines the feasibility of compassion-based DEI training by showing whether the training will increase compassion and empathy and reduce stigmatizing thoughts and modern prejudice. It is important to develop new innovative methods for DEI work, as the effects have often been lacking in prior research (Paluck et al., 2021; Hsieh et al., 2022).

## Discrimination in the Swedish context

Discrimination is defined as behavior patterns that have disadvantageous outcomes for people belonging to a marginalized social group compared to a majority group (Kite and Whitley, 2016). Despite the anti-discrimination laws in Sweden (Discrimination Act 2008: 567, 2017:1128), prejudice and discrimination still cause societal problems. The Swedish Equality Ombudsman, a governmental agency combating discrimination, analyzed 1,800 individual complaints of perceived discrimination, revealing extensive discrimination (Kumlin, 2014). These reports encompass personal experiences across various sectors, including employment, education, healthcare, public services, and access to goods and services.

Reports indicate that ethnic Swedes exhibit significantly higher salaries compared to non-ethnic Swedes (individuals born abroad or with foreign-born parents). Furthermore, ethnic Swedes experience more frequent promotions and greater workplace support from mentors and colleagues (Wolgast and Wolgast, 2021; Wolgast et al., 2018b). Recruitment studies demonstrate that applicants with Arabic names face a lower likelihood of selection compared to those with Swedish names (Bursell, 2012; Wolgast et al., 2018a). What are the mechanisms behind the prejudice that results in such racist discrimination?

## Prejudice and emotions

Social psychology presents mechanisms operating both cognitively and emotionally that explain the origins and maintenance of racism and other forms of discrimination in society. Both cognitive and emotional processes in the development of prejudice against specific groups often emerge through socialization processes and social categorization that associate certain groups with negative stereotypes (Cuddy et al., 2009; Talaska et al., 2008). Moreover, it has been proposed that negative prejudice directed toward stigmatized groups is deeply ingrained within the culture (Devine, 1989), and indeed, in many cultural contexts, prevailing norms tend to advantage non-stigmatized dominant social groups (Akrami and Ekehammar, 2005).

Consequently, prejudiced attitudes are frequently so thoroughly internalized that they become activated automatically upon encountering or even thinking about a member of a stigmatized group (e.g., Greenwald et al., 1998). Such thinking can be referred to as “inflexible stigmatizing thoughts,” which involves individuals’ lack of awareness of how their stigmatizing thoughts influence their emotions and behavior (Levin et al., 2014).

Empirical evidence indicates that the emotional component serves as a significant impetus for prejudiced actions and stigmatizing thoughts (Crandall and Eshleman, 2003), mediating the relationship between stereotypes and discrimination, often operating unconsciously (Cuddy et al., 2009; Stanley et al., 2008; Talaska et al., 2008). Research suggests that individuals frequently base their judgments on emotional experiences rather than facts (Talaska et al., 2008). Despite the intellectual understanding that prejudice is unethical, it is challenging to exert conscious control over automatic emotional responses (Stephan, 2014; Kite and Whitley, 2016). Covert modern prejudice manifests even among individuals who espouse humanistic values, express egalitarian viewpoints, and perceive themselves as open-minded. However, in ambiguous situations, these individuals may exhibit discriminatory behavior (Massey et al., 2013). For instance, they might undermine equality by denying the existence of discrimination against certain social groups or expressing disapproval of compensatory support initiatives (Akrami et al., 2000). Research indicates that modern prejudice impacts discrimination across various domains, including job interviews, mentorship, and legal proceedings (Massey et al., 2013).

There are different forms of interventions targeting less conscious prejudice, including brief “light-touch” methods (e.g., counter-stereotypical training) that demonstrate short-term changes in implicit preferences as measured by the Implicit Association Test—IAT (Forscher et al., 2019; Lai et al., 2016). However, these effects often do not persist beyond one day (Lai et al., 2016) and are primarily observed in implicit measures with an unclear relationship to explicit prejudice or behavior (Blanton et al., 2015). More promising habit-breaking interventions utilizing IAT results to give participants feedback on their prejudice level, followed by training in bias-reduction strategies, have demonstrated both prejudice reduction and behavioral effects in student samples (Forscher et al., 2017). Nevertheless, addressing the defensiveness that might arise from IAT feedback (Howell et al., 2017) and mitigating feedback derogation are crucial in DEI programs. Research has shown that rejection of feedback (on individuals’ prejudice) may increase negative intergroup behavior (Howell et al., 2017). Given the uncertain outcomes of many interventions and the need to manage defensive reactions, the continued development of novel methods, such as compassion training, is necessary to combat less conscious prejudice and inflexible stigmatizing thoughts.

## Third wave behavior philosophy

Clinical psychotherapists have a long tradition of assisting clients in processing negative emotions and handling defensive and avoidance behaviors. Furthermore, third-wave behavioral therapies use interventions targeting processes that aim to disturb language processes (e.g., arbitrary biased associations underlying thoughts) to induce inner contextual change. This shift can help individuals reach awareness of their emotions, as well as their thoughts about others and the emotional reactions they evoke, thereby increasing the probability of behavior change (Hayes et al., 2001). This internal contextual change is facilitated by developing a compassionate, safe system for self- and other-related actions. A compassionate mindset can be cultivated through therapy (Gilbert, 2014) and beyond (Gilbert and Choden, 2014) via mindfulness and experiential interventions promoting self-compassion, compassion for others, and receptivity to compassion. The compassionate self (safe system) in CFT is a regulatory system characterized by sensitivity to emotions; understanding the origins of suffering; courage to alleviate it; warmth, sympathy, empathy, and security; and a non-judgmental approach. This system needs to interact with the threat and drive systems to mitigate the impact of aversive emotions. Moreover, being compassionate means increasing individuals’ ability to feel connected to other people (Gilbert, 2014; Gilbert and Choden, 2014).

Gilbert (2017) posits flexibility, encompassing awareness of thoughts/feelings as reactions and self-control for goal-oriented behavior (Hayes et al., 1999), are essential to compassion. Moreover, Trich et al. (2014) have formulated an integrative concept that aligns compassion and flexibility, namely “Compassionate flexibility,” and it refers to the ability to not avoid aversive reactions, to be flexible, and to consciously choose compassionate actions.

CFT employs experiential exercises to cultivate processes such as mindfulness, attentional control, acceptance, and behavioral change. In CFT and mindful compassion approaches, the compassionate self is actively developed not only as a regulatory system but also as a foundation for values, guiding prosocial behavior. Compassion for others may reduce the need to compete with others, including defending social status and resource acquisition (Kolts, 2016). Research indicates that compassionate states, characterized by security and tranquility, are associated with a decreased perception of others as threatening (Mikulincer and Shaver, 2017) and less negative outgroup attitudes (Vu and Rivera, 2023), suggesting the utility of compassion training in DEI initiatives. How can compassion function to reduce prejudice, and inflexible stigmatizing thoughts?

## The path from compassion to reduction in inflexible thoughts

We propose a theoretical model for how compassion can lead to a reduction in prejudice and stigmatizing thoughts, incorporating three core mechanisms: (1) *Emotional Regulation* activating the parasympathetic ‘soothing system’ (CFT) reduces threat-based emotions (e.g., fear, disgust) underlying prejudice (Gilbert, 2009; Mikulincer and Shaver, 2017), promoting warmth toward stigmatized groups and reducing stigmatizing thoughts; and (2) *Perspective-Taking and Empathic Concern* enhancing affective and cognitive empathy (Dovidio et al., 2017) reduces dehumanization and fosters prosocial motivations by enabling individuals to understand and respond to others’ experiences. (3) *Compassionate Flexibility* involves noticing and accepting discomforting thoughts and emotions (e.g., implicit biases) though not avoiding them Trich et al. (2014).

This facilitates reflective and inclusive engagement, contrasting with automatic, prejudice-driven reactions. It may also mitigate defensiveness within DEI contexts, thereby fostering the acknowledgment of inequalities.

Drawing on the Stereotype Content Model (Cuddy et al., 2009), we acknowledge that prejudice may operate through varied psychological mechanisms. We suggest that compassion training is posited as particularly effective for addressing prejudice driven by fear, disgust, or dehumanization and that shifting affective responses can yield significant attitudinal change. For prejudice rooted in perceived status threat or competition (e.g., envy), alternative strategies such as cooperative goals or structural equity interventions may be more impactful. This nuanced approach suggests that compassion training is most suitable for emotionally driven forms of modern prejudice and for cultivating inclusive behavior by reducing emotional avoidance and defensiveness. What DEI programs fully or partly integrate mechanisms grounded in empathy and compassion?

## Existing programs with compassion and empathy content

Conventional theories behind anti-discrimination/DEI programs fall into three categories (Aboud, 2008): *intergroup contact* (Pettigrew and Tropp, 2006), *awareness-raising instruction* on equality benefits and discrimination issues, and *social-cognitive interventions* (Hsieh et al., 2022; Paluck et al., 2021) encompassing cognitive acceptance, recategorization, mindfulness, and perspective-taking. Though all three may indirectly evoke empathy and compassion—intergroup contact potentially fostering compassion and empathy, awareness raising potentially increasing sympathy, and social-cognitive approaches furthering compassion and perspective taking—few DEI interventions utilize compassion as the primary mechanism.

The most common training goal for self-compassion or compassion interventions is to increase mental health, and several studies have shown positive effects on compassion for others, empathy, and mental health (e.g., Neff and Germer, 2013; Lutz et al., 2008).

Concerning using compassion for anti-discrimination/DEI work, there are a few studies describing effective programs. One study with an organizational sample combining compassion with flexibility training (Wolgast et al., 2024) found effects on compassion, empathy, and stigmatizing thoughts. A second organizational DEI training with one compassion exercise demonstrated prejudice reduction (Ilanius Göransson et al., 2025). Another study with 3^rd^-5^th^ grade children combined compassion and mindfulness to foster prosocial behavior with promising results, including long-term effects on reduced affective prejudice, negative stereotyping, and increased willingness for intergroup contact (Berger et al., 2018). Apart from these, there are also very brief interventions for adults (10 min) with compassion and mindfulness exercises demonstrating effects on decreased intergroup anxiety, increased positive intentions toward stigmatized groups (Parks et al., 2014), and reduced discriminatory behavior (Lueke and Gibson, 2016). Finally, a study of a 6-session intervention of compassion meditation found a change in implicit but not explicit attitudes (Kang et al., 2014).

Consequently, training in compassion seems to be a promising method to use to improve DEI because compassion training includes both exercises aimed at increasing compassion for others and individuals’ intentions to actively relieve other people’s suffering (Gilbert, 2009), highlighting the role of compassion in fostering interpersonal relations (Gilbert and Choden, 2014). In sum, even if there are studies showing that mindfulness interventions, including empathy and compassion training, have some effects on prejudice and stigmatizing thoughts, most of them have not measured the effects of empathy and compassion and have not been designed to function as DEI training.

## The current study

Prior research thus suggests that compassion training can enhance positive constructs such as compassion and empathy, which have also been shown to be linked to reduced negative attitudes toward outgroups (Levin et al., 2016; Vu and Rivera, 2023). Therefore, compassion is likely to mitigate stigmatizing thoughts and prejudice (Gilbert et al., 2017; Trich et al., 2014). This pilot study represents an initial phase in the development of a concise (2-h) group intervention grounded in CFT exercises designed for organizational implementation to enhance Diversity, Equity, and Inclusion (DEI) and diminish discrimination. The current research contributes to the literature by advancing knowledge regarding the development and measurement of a compassion-based DEI program.

First, the study investigates the relationships between the measured variables to determine whether compassion and empathy are negatively associated with stigmatizing thoughts and modern racist attitudes as indicators of the training’s potential for change. The primary aim of this study is to examine whether compassion training increases compassion to others and empathy and decreases stigmatizing thoughts and modern racist attitudes. We assume that there will be (H1) a negative link between compassion/empathy and stigmatizing thoughts/modern racist attitudes. We further hypothesize that compassion training in the group will (H2) increase participants’ compassion for others, (H3) empathy, (H4) reduce stigmatizing thoughts, and (H5) modern racist attitudes in the experimental group versus the comparison condition.

## Method

### Sample

The participants were 33 Swedish non-psychology university students above 18 years of age. The recruitment of participants without a background in psychology was intended to mitigate the potential influence of pre-existing familiarity with psychological methodologies, such as compassion training, on their engagement with the intervention. There were 16 participants in the comparison group (11 women, 5 men) and 17 participants in the experimental group (14 women, 3 men). The average age in the comparison group was 26.8 years (range 19–52), and that in the experimental group was 23.7 years (range 19–37). The comparison participants conducted their studies in Social Sciences (4), Humanities (5), and Natural Sciences (5), and 2 were labeled “other,” involving participants who did not state what they studied or worked on. The experimental participants conducted their studies in Social Sciences (6), Humanities (1), Natural Sciences (4), and 6 were labeled “other,” involving participants who did not state what they studied or stated that they worked or were unemployed.

### Instruments

#### Compassion to others

Compassion To Others (CTO) was constructed for the present study to measure compassion to others and contains 26 items and 5 subscales (see Appendix). Participants were instructed to answer how frequently they behaved in accordance with the statements. A five-point Likert scale with 1 referring to “almost never” and 5 to “almost always” was used. Examples of items are “When others feel alone in their suffering, I try to help them see their suffering as something human” (perspective-taking/common humanity), “I tend to get caught up in and ruminate on other people’s problems” (over-identification), “I become critical when others do not perform as I expect” (tolerance/intolerance), “I find it difficult to relate to others when they talk about feelings” (lack of empathy), and “I try to face the suffering of others with kindness” (kindness).

The development of the CTO is grounded in the theoretical framework of the CFT (Gilbert, 2009), the three-flow model of compassion (Gilbert, 2017), and relevant psychometric instruments such as Neff (2003) Self-Compassion Scale and Gilbert et al. (2017) Compassionate Engagement and Action Scales. The aim was to construct an instrument that captures both emotional and behavioral dimensions of compassion directed toward others, particularly in response to others’ suffering. The initial item pool was created by the research team, drawing directly on theoretical constructs and items from existing compassion scales, including a comprehensive literature review and integration of definitions by Strauss et al. (2016) and Gilbert et al. (2017). Items were grouped into five theoretically derived sub-constructs: perspective-taking, over-identification, tolerance, empathy, and kindness. Items were reviewed for face validity by experts in compassion-based therapy and diversity training. The scale was piloted with psychology-informed individuals and lay participants to assess item clarity and accessibility. Following initial data collection, an exploratory factor analysis (EFA) was conducted to examine the underlying structure of the CTO. Items that did not load clearly onto the factors were removed or revised. The final version contained five subscales, each reflecting a distinct component of compassion. Internal consistency for each subscale was found to be acceptable to good: perspective-taking/common humanity (*α* = 0.83), over-identification (*α* = 0.78), tolerance/intolerance (*α* = 0.85), lack of empathy (*α* = 0.72), and kindness (*α* = 0.78). The overall internal consistency of the full scale was *α* = 0.82, indicating good reliability.

#### Empathy

Empathy was measured with the Interpersonal Reactivity Index (IRI), which consists of 28 questions. It is based on Davis’s (1983) four-factor model, which describes empathy from two emotional aspects, empathic concern and personal distress, as well as two cognitive aspects, perspective-taking and fantasy. All four factors described some form of responsiveness to others’ signals. Empathic concern describes the ability to sympathize with the difficulties of another person, and personal distress describes the individual’s experience of discomfort when another person is suffering. Personal distress was reverse-coded in the present study, as the training did not intend to increase personal distress; rather, the contrary lowered it. The cognitive aspects, perspective-taking, and fantasy describe individuals’ abilities to see things from another person’s perspective and the ability to imagine how fictional people think and feel (Davis, 1983). Internal reliability showed Cronbach’s alpha between *α* = 0.71 and 0.77, and test–retest reliability was good (Davis, 1983). In this study, internal reliability was *α* = 0.85.

#### Stigmatizing thoughts

Stigmatizing thoughts were measured with the Acceptance and Action Questionnaire— Stigma (AAQ-S). This scale measures psychological inflexibility in relation to stigmatizing thoughts (Levin et al., 2014). Levin et al. conceptualize stigma as a general tendency to value and discriminate against individuals based on their social group affiliation. It has two subscales: psychological inflexibility and psychological flexibility. The 20 items are scored on a seven-point Likert scale from 1 = “not at all correct” to 7 = “completely correct.” Examples of items are Inflexibility: “My biases and prejudices affect how I interact with people from different backgrounds.” Flexibility: “My negative thoughts about others are never a problem in my life.” Analyses have indicated that the AAQ-S psychological inflexibility and flexibility subscales, as well as the combined total score, correlate with other measures of psychological flexibility as well as stigma and are more predictive of stigma than a general measure of psychological flexibility. The scale also correlated significantly with other scales measuring stigmatizing thoughts, prejudice, and psychological flexibility (Levin et al., 2014). The internal consistency reported in (ibid.) was 0.84, and Cronbach’s alpha for the present study was 0.84.

#### Modern racist attitudes

The Modern Racial Prejudice Scale (MRPS; Akrami et al., 2000) was used to measure modern racist attitudes. It is a self-assessment scale and consists of nine questions with a five-point Likert scale where 1 is “not correct at all,” and 5 is “exactly correct.” Examples of questions are “There have been enough efforts for the unemployed immigrants” and “Discrimination against immigrants is no longer a problem in Sweden.” The scale correlated significantly with other validated measures such as Conservatism, Modern Sexism, Classical Sexism, and SDO (Akrami et al., 2000). The internal reliability was *α* = 0.82 (Akrami et al., 2000). In the current study, the scale showed an internal reliability of *α* = 0.89.

### Procedure

One week prior to the intervention, participants completed the pre-test survey and provided informed consent before undergoing randomized assignment. The experimental group received 2-h of compassion training, while the comparison group attended lectures on prejudice and discrimination for an equivalent duration.

### The intervention

Although compassion-based interventions sometimes span several sessions, the present study intentionally used a brief, 2-h format. This decision was informed by recent calls in the literature to design scalable interventions suited for organizational contexts, where time constraints can hinder implementation (Kirby et al., 2017). This pilot study tests the feasibility and preliminary effects of a brief, single-session intervention that could later be adapted for workplace training and anti-discrimination efforts. Although more extensive interventions may yield stronger effects, a brief intervention may still offer meaningful psychological benefits.

#### Description of the experiment group condition

The experiment training included mindfulness and visualization exercises to practice compassion and kindness to others, instruction of theoretical models of the three regulating systems (drive-, threat-, and safety-systems), discussion and reflections of the participants’ experiences of these systems, and exercises to increase the safety system (see Table 1). The focus of the intervention was primarily to strengthen participants’ ability to feel compassion for others. The training was based on principles from the CFT and structured as a single session of 2 × 45 min with a 15-min break. Prior to implementation, the intervention was pretested with individuals knowledgeable in psychology, as well as lay individuals, and modifications were made to optimize clarity and accessibility. Each exercise was succeeded by a group discussion, allowing participants to articulate their reflections.

**Table 1:** Overview of the compassion training intervention.

Phase	Content	Purpose
1. Introduction and grounding	*- Breathing anchor (mindfulness)* *- Lemon imagination exercise*	*- Increase present-moment awareness as a foundation for behavioral change* *- Demonstrate the impact of thoughts on emotions and bodily sensations*
2. Psychoeducation: emotion Regulation systems	*- Presentation of the three affect regulation systems: Threat, Drive, and Soothing* *- Personal reflection on time spent in each system* *- Guided visualization of walking to work/school in threat vs. soothing system*	*- Provide a theoretical framework for emotional responses* *- Strengthen awareness of internal states and how they influence perception and action*
3. Building access to the soothing system	*- Loving-kindness visualization directed first toward a loved one, then toward oneself* *- Compassion for someone suffering: reflection on helpful actions* *- Compassion for a difficult person: visualizing similarity, exploring needs, writing a compassion letter*	*- Gradually strengthen compassion skills, beginning with familiar and moving toward more challenging targets* *- Encourage perspective-taking and emotional regulation*
4. Addressing barriers and real-life application	*- Group discussion: barriers to feeling compassion (for self and others)* *- Short film of a morally ambiguous person, followed by reflection* *- Final reflection: how to apply compassion in everyday life*	*- Normalize inner resistance to compassion* *- Support integration of compassion into participants’ everyday behavior and mindset*

The table outlines the structure, content, and purpose of each phase in the compassion training intervention, which was designed to cultivate compassion for others through experiential exercises, reflection, and group discussion.

### Description of the comparison condition

The comparison group underwent an instructional and discussion-based session, adapted from Lundgren and Hasselberg (2019). This condition was delivered in two 45-min blocks with a 15-min intermission and was specifically designed to mirror standard workplace training on prejudice and discrimination. The lecture included:

- Factual information on prejudice and discrimination, sourced from the Swedish Equality Ombudsman (DO).
- Psychological theories explain prejudice and how social norms influence individuals.
- Two short films illustrate the prejudice.
- Group discussions, in which participants reflected on prejudice and discrimination in higher education.

Thus, the session integrated passive elements (lecture and film) with active elements (group discussion), facilitating participants’ engagement with the material through both cognitive processing and reflection.

### Data analysis

The data were screened for outliers, and four outliers were identified. These outliers were subsequently adjusted by recording them to the next-highest value within the boxplot. (Tabachnick and Fidell, 2007). Normality, covariance equality (Box’s), and error variance equality (Levene’s) were investigated, and no deviations were found. Skewness and kurtosis were between −1 and +1 for all variables (Hair et al., 2022), and the Shapiro–Wilk test of non-normality was non-significant for all measures. An independent t-test was used to verify that randomization yielded comparable groups, and the results indicated no significant differences between the conditions at the pre-test measures (*p* > 0.05). To analyze the effects of pre- and post-measurements of the different conditions (experimental and comparison), mixed ANOVAs were performed for the outcome variables. Partial Eta-Squared (*η_p_^2^*) is the effect size used to describe how strong the connection was between independent and dependent variables, where a weak effect was *η_p_^2^* > 0.01, a moderate effect was *η_p_^2^* > 0.06, and a strong effect was *η_p_^2^* > 0.14 (Cohen, 1988). Despite the relatively small sample size (*N* = 33), a mixed-design ANOVA was deemed appropriate for the analysis because the data met the assumptions of normality. ANOVA has been shown to be robust to moderate violations of normality and homogeneity of variances, particularly when group sizes are roughly equal (Blanca et al., 2017; Schmider et al., 2010). Moreover, parametric methods, such as ANOVA, remain preferable to non-parametric alternatives when assumptions are met, as they offer greater statistical power and more straightforward interpretability of interaction effects (Field, 2013). Effect sizes are reported alongside *p*-values to provide an estimate of the magnitude of observed effects, as recommended in statistical reporting guidelines (Lakens, 2013).

### Ethics statement

This study, including the experimental protocol, was approved by the Swedish Ethical Review Authority (Dnr: 2018/567). All participants provided informed consent and were informed that they could withdraw from the study at any time. We also considered the potential emotional impact of reflecting on participants’ own and others’ suffering. Participants were reminded that the training was not a test situation but an opportunity to explore and practice. Participants were clearly informed that group sharing was voluntary, and facilitators were attentive to emotional reactions and framed compassion as a skill under development.

## Results

Table 1 describes means, standard deviations, and the associations between the four measures of the sample.

As stated in H1, significant negative correlations were found between compassion/empathy and stigmatizing thoughts/modern racist attitudes. Compassion had a large effect in relation to stigmatizing thoughts and a medium effect in relation to modern racist attitudes, whereas empathy had a large effect in relation to both stigmatizing thoughts and modern racist attitudes. H1 was thus confirmed.

The pretest between-condition analyses to ascertain equal groups indicated no significant differences between the groups for any of the measures before the training, which means that the groups could be considered equal.

### Pre- and post-measurement results for the training (H2-H5)

Table 2 presents means and standard deviations for results at pre- and post-measurements for the outcome measures of the two conditions: compassion to others (CTO), empathy (IRI), stigmatizing thoughts (AAQ-S), and modern racist attitudes (MRPS).

**Table 2:** Mean, standard deviation, and correlation for the measures at the pretest.

Measures	M (SD)	CTO	IRI	AAQ-S
Compassion (CTO)	98.1 (10.9)			
Empathy (IRI)	98.21 (12.25)	0.65		
Stigmatizing thoughts (AAQ-S)	61.27 (15.58)	−0.73	−0.56	
Modern Racism (MRPS)	14.7 (5.03)	−0.36	−0.55	0.39

The mean per item on MRPS was 1.63 in the present study as compared with Akrami et al. (2000), who found 1.95 in their Swedish sample.

A mixed between-within-subjects ANOVA was used to examine whether the compassion training increased compassion for others, empathy, stigmatizing thoughts, and modern racial attitudes by comparing the effects of the two conditions.

The results for compassion showed a strong and significant interaction effect between condition and time [*F*(1, 31) = 6.1; *p* = 0.02; *η_p_^2^* = 0.16]. The results corroborated H2, meaning that the compassion training group’s increase in compassion was greater than that of the comparison group (Table 3).

**Table 3:** Means and standard deviations in two conditions at pre- and post-measurement.

Measures	EXPERIMENT (*N* = 17)		COMPARISON (*N* = 16)	
	Pre-M (SD)	Post-M (SD)	Pre-M (SD)	Post-M (SD)
Compassion	96.00 (10.24)	99.65 (9.62)	100.44 (13.32)	98.94 (12.29)
Empathy	95.35 (13.45)	103.59 (9.50)	101.25 (10.40)	103.69 (10.10)
Stigmatizing thoughts	63.41 (15.01)	60.59 (11.41)	59.00 (16.33)	62.06 (17.44)
Modern racism	15.12 (4.96)	14.12 (4.04)	14.25 (5.22)	14.25 (3.36)

The results for empathy demonstrated a medium and significant interaction effect between condition and time, *F*(1, 31) = 5.53, *p* = 0.03, and *η_p_^2^* = 0.15. Thus, the results provided support for H3, meaning that the compassion training group’s increase in empathy was greater than the comparison group.

The result for stigmatizing thoughts showed a tendency for an interaction effect, *F*(1, 31) = 2.73, *p* = 0.06, *η_p_^2^* = 0.11. The results provided no definite confirmation of H4, even though there was a tendency in the predicted direction that the compassion training seemed to have some reducing effects on stigmatizing thoughts in the compassion training group in comparison with the lecture group.

### Modern racist attitudes H5

The result for modern racist attitudes showed that there was no significant interaction effect, *F*(1, 31) = 0.67, *p* = 0.42, and *η_p_^2^* = 0.02, nor any main effects for the conditions. The results thus provided no support for H5, as the experimental group’s reduction in modern racist attitudes was similar to the comparison group’s.

## Discussion

This pilot study explored whether a brief group-based compassion training intervention could influence compassion, empathy, stigmatizing thoughts, and modern racist attitudes. Though the small sample size and brief duration limit the strength of conclusions, the findings offer preliminary insights into the potential psychological processes involved in prejudice reduction. As an exploratory investigation, these results provide early support for the feasibility of integrating compassion-based exercises into diversity, equity, and inclusion (DEI) interventions. As a pilot study, these findings serve as a preliminary step in developing scalable interventions that can be implemented in real-world organizational settings. The significant effects on compassion and empathy, alongside a tendency for a decrease in stigmatizing thoughts, suggest that even a short intervention can produce measurable changes.

The first hypothesis concerned the relations between the measures, for which negative relations were expected between compassion/empathy and stigmatizing thoughts/modern racist attitudes, and was supported. These were the basic assumptions behind the study, which were met, indicating that it could be beneficial to practice compassion and empathy to decrease stigmatizing thoughts and racial attitudes. Such associations have been noted in earlier studies too, between empathy and prejudice (Beelmann and Heinemann, 2014; Miklikowska, 2018; McFarland, 2010; Bergh and Akrami, 2016), between empathy and stigmatizing thoughts (Levin et al., 2016), and between compassion and prejudice (Berger et al., 2018). Even if behavioral outcome was not measured in the present study, previous studies have shown that high empathy generally predicts both lower prejudice and less discriminatory behavior (Galinsky and Moskowitz, 2000; Kite and Whitley, 2016; Levin et al., 2016) and that empathy and compassion can lead to prosocial behavior (Preckel et al., 2018). These studies support the ambition to target both compassion and empathy in order to reduce discriminatory behaviors and promote prosocial behaviors.

The second hypothesis—that compassion training would increase compassion toward others—was supported by a significant group × time interaction, with a large effect size. This suggests that even a brief intervention may influence compassion-related processes, although further replication in larger samples is necessary. DEI programs that train compassion abilities in order to decrease prejudice/discrimination are scarce even though there are a few with promising results that found positive effects of mindfulness interventions (Berger et al., 2018; Lueke and Gibson, 2016; Parks et al., 2014; Wolgast et al., 2024). However, the current study did not only measure racist attitudes and stigmatizing thoughts, but we also measured the effects on compassion to others, corroborating that compassion indeed changed, as might be a limitation in most earlier studies.

The third hypothesis, that compassion training would increase empathy compared to the instruction group, was also supported, and the effect was medium-sized. This result is in line with previous literature that shows that empathy will also increase when compassion is practiced and might be another important process in DEI and anti-discrimination work (Dovidio et al., 2017; Talaska et al., 2008).

The fourth hypothesis, that compassion training would reduce stigmatizing thoughts relative to the comparison group, was not statistically supported (*p* = 0.06). Although the observed effect size (*η_p_*^2^ = 0.11) suggests a potential difference, this finding must be interpreted with caution and cannot be considered as evidence of an effect in this underpowered, exploratory context. In comparison with prior literature, two studies found a significant reduction in stigmatizing thoughts as a result of DEI training with organizational samples (Wolgast et al., 2024; Ilanius Göransson et al., 2025). These interventions involved a combination of compassion exercises and other approaches, with a substantial compassion part in Wolgast et al.’s study and one compassion exercise in Ilanius Göransson et al. (2025) study.

Researchers have argued that compassion may function as a process that reduces stigmatizing thoughts, especially through what Trich et al. (2014) called “compassionate flexibility.” Though not directly measured in our study, we propose that this process may serve as an intermediary mechanism for how compassion training can lead to change in inflexible stigmatizing thoughts and some forms of prejudice. Future research should develop a scale to capture compassionate flexibility to explore this proposition further.

Moreover, the suggested theoretical framework distinguishes between different forms of prejudice by recognizing the diverse emotions connected to them. Prejudice can stem from fear and disgust, as well as from envy and perceived threat to group status. Compassion training may be more effective in reducing the former by transforming threat-based affective responses through emotional regulation and empathy enhancement. For the latter—where prejudice is rooted in competitiveness or perceived injustice—other interventions may be necessary. This differentiation aligns with the Stereotype Content Model (Cuddy et al., 2009) and allows for a more strategic and context-sensitive application of DEI tools.

The fifth hypothesis, that compassion training would reduce modern racist attitudes, was not supported. One possible explanation is the floor effect, as baseline levels were already low, particularly given the young student sample. This limitation, along with the small sample size and brief intervention length, may have constrained the possibility of observing change in this outcome. When comparing Akrami et al. (2000) means for Swedish participants, the present study’s participants had substantially lower means from the beginning. Another explanation for why little change was achieved in prejudiced attitudes may be that when working with compassion and empathy, these processes do not always contribute to changes in thoughts but rather contribute to an awareness of negative thoughts and feelings that, for instance, prejudice may evoke. Through mindfulness and experiential interventions, participants might change their relation to the contents of their thoughts (Hayes et al., 2001), and in the current study, it may refer to the fact that they do not respond with feelings of fear and negative actions because of their negative thoughts. The change of relation to thought contents might thus result in a more compassionate and empathic approach to others and possibly fewer responses to stigmatizing thoughts. With these arguments in mind, it might not be surprising that the present study did not show any change in racist attitudes; thus, the measurement for racist attitudes used might have been a less relevant tool. In contrast to this assumption, Berger et al. (2018) showed that it was possible to change both emotional and cognitive components of prejudice as measured with explicit attitude measures. Their study was, however, more extensive (stretched across 24 weeks), possibly implying that a certain study duration might be needed to achieve prejudice reduction, and apart from compassion training, they also trained in prosocial skills and coping strategies. Some mindfulness and compassion studies on adults have also found decreased implicit prejudice (Kang et al., 2014) and explicit attitudes (Parks et al., 2014). Future studies with similar interventions as ours could elaborate on the present intervention by adding sessions and including more mindfulness training and behavior training, such as training in prosocial behavior and roleplay, to achieve prejudice reduction.

Interestingly, the comparison group, which received lectures on prejudice, showed a slight decrease in compassion for others and an increase in stigmatizing thoughts. One possible explanation is that being exposed to injustice-related content without tools for emotional regulation (such as those provided in the compassion training) may lead to defensiveness (Howell et al., 2017), guilt, or resistance—especially if participants feel implicitly judged or implicated. This has been discussed in previous research on DEI backlash effects (Chang et al., 2019; Howell et al., 2017). These findings suggest that merely increasing awareness of discrimination may not be sufficient and could, in some cases, increase emotional resistance if not accompanied by self-reflective and compassion-based practices.

### Limitations and future research

This pilot study was conducted to ascertain that the training and measures worked. For this reason, a group of university students (from disciplines other than psychology) were recruited; however, we did not ask whether the students had prior experience with mindfulness, which also could have influenced the result. In addition, although the data met the assumptions for parametric analysis, the relatively small sample size (*N* = 33) introduced important statistical limitations. Small samples are particularly vulnerable to Type II errors—that is, failing to detect true effects—especially for interaction effects or effects of modest size (Maxwell et al., 2008). Though the Type I error rate is nominally controlled by the alpha threshold (e.g., *p* < 0.05), small samples also increase estimation variability and sampling error, which can compromise the stability of results and occasionally inflate the risk of false positives (Button et al., 2013). To contextualize the strength of the findings, effect sizes are reported alongside *p*-values. A *post hoc* power analysis using the observed effect sizes (partial η^2^ = 0.02–0.16) indicated that the achieved power ranged from 0.13 to 0.68, further underscoring the limited sensitivity of the study. These results support interpreting the present study as a pilot investigation, and replication with larger, adequately powered samples is recommended to assess the robustness and generalizability of the findings (Lakens, 2013).

In addition, given the exploratory nature of this study and the small number of tests (*n* = 4), we report uncorrected *p*-values, in line with methodological recommendations that caution against overly conservative corrections in such contexts (Gelman et al., 2012; Rothman, 1990). Strict adjustments such as the Benjamini-Hochberg (BH) procedure can reduce Type I errors but may inflate Type II errors, potentially masking effects worth further investigation (Feise, 2002). In our analysis, two results (*p* = 0.02 and 0.03) met the conventional threshold for significance. However, under BH correction, their *q*-values were 0.06—just above the 0.05 cutoff. Although not formally significant after correction, we interpret these as preliminary, hypothesis-generating findings that warrant replication. This approach aligns with the goal of exploratory analysis: to identify possible effects, not to draw confirmatory conclusions (Cumming, 2014).

The brevity of the intervention is also a limitation of the present study. Though some compassion-based diversity interventions involve multiple sessions over several weeks (Berger et al., 2018; Kang et al., 2014), the present study tested the potential of a 2-h session. The rationale for this choice was to explore the feasibility of implementing brief interventions in organizational contexts. In contrast, other mindfulness and compassion studies have been very brief (10 min) and still found effects on discrimination (Lueke and Gibson, 2016) and explicit attitudes (Parks et al., 2014). Although effects were detected in these and the present study, it remains uncertain how long-lasting or deep these effects are. Future studies should compare short versus extended formats to determine optimal intervention dosage.

Measurement limitations must also be acknowledged. Though changes in compassion and empathy were clearly observed, the racist attitude measure did not show any effects, and the reduction in stigmatizing thoughts only approached significance. Other measures could be relevant to include in future research—particularly behavioral outcomes, which are the ultimate goals of DEI programs. Additionally, future studies should explicitly prioritize the development and validation of a psychometric instrument to measure *compassionate flexibility*, a construct central to our theoretical framework. Though this study draws on Trich et al. (2014) conceptualization of compassionate flexibility—as the capacity to remain present with aversive thoughts and emotions while choosing compassionate action—there is currently no dedicated, validated scale to empirically capture this mechanism. Developing such a measure would enable a more precise evaluation of whether compassion-based interventions genuinely foster this quality and how they mediate changes in prejudice-related outcomes. Including compassionate flexibility as a measurable outcome would also help differentiate the effects of compassion training from broader constructs such as general empathy or mindfulness. Future studies should explore the longitudinal stability of compassionate flexibility and its role in buffering defensiveness and resistance in DEI training settings.

## Conclusion

This pilot study provides preliminary evidence that a brief, 2-h compassion training may influence self-reported compassion and empathy. Though the findings are exploratory, they offer a basis for future research on compassion-based components in DEI training as feasible roads for employers who want to implement positive intergroup relations, diversity, equity, and inclusion in their organizations.

## Data Availability

The raw data supporting the conclusions of this article will be made available by the authors, without undue reservation.
