# Supplementary material for: Impact of group training on compassion, empathy, and stigmatizing thoughts: a diversity, equity, and inclusion pilot RCT
Source: Front Psychol. 2025 Jun 20;16:1547645. doi: 10.3389/fpsyg.2025.1547645 (PMC12231507; doi:10.3389/fpsyg.2025.1547645)
Supplement: Supplementary file 1 [file Supplementary_file_1.pdf]

Appendix

Item List for Compassion to Others (CTO)

1. I am disapproving and judgmental of others' mistakes and shortcomings. T/IT-r
2. I tend to ruminate and dwell on other people's problems. OI-r
3. I am hard on others and set high expectations, even when they are struggling. T/IT-r
4. When I notice traits in others that I dislike, I devalue them. T/ITr
5. I am kind to others when they are suffering. K
6. When others are upset, I become filled with their emotions. OI-r
7. I am tolerant of others' mistakes and shortcomings. T/IT
8. I can handle other people's difficulties without becoming overwhelmed myself. OI
9. I don't know how to help others when they are having a hard time. LE-r
10. I can share and support others in their suffering without becoming overwhelmed. OI
11. When others fail, I feel contempt for them. T/IT-r
12. When others fail, I criticize them. T/IT-r
13. I find it hard to relate to others when they talk about emotions. LE-r
14. I criticize others when they don't succeed. T/IT-r
15. I try to meet others' suffering with kindness. K
16. I believe that all human suffering deserves to be met with kindness. K
17. When others feel alone in their suffering, I try to help them see it as something human. PT/CH
18. When others feel alone in their negative emotions, I try to help them see it as something human. PT/CH
19. I tend to make others' suffering my own. OI-r
20. I criticize others for their shortcomings. T/IT-r
21. I become critical when others don't perform as I expect. T/IT-r
22. When others are upset, I don't know what to do. LE-r
23. When others show negative emotions, I try to respond to them in the moment. PT/CH
24. When others show negative emotions, I try to meet them with warmth. PT/CH
25. When I see someone displaying negative emotions, I try to relieve and ease them. PT/CH
26. When others experience negative emotions, I feel helpless. LE-r

Subscales: T/IT = Intolerance (items reverse coded for total score, except item 7), OI = Overidentification (reverse coded for total score, except item 8,10); K = Kindness; LE = Lack of Empathy (reverse coded for total score), PT/CH Perspective-Taking/Common Humanity; r = Reverse coded for the total score for compassion.
